# A Sensitive and Quantitative mKeima Assay for Mitophagy via FACS

**DOI:** 10.1002/cpcb.99

**Published:** 2019-12-26

**Authors:** Chunxin Wang

**Affiliations:** ^1^ Biochemistry Section, National Institute of Neurological Disorders and Stroke National Institutes of Health Bethesda Maryland

**Keywords:** FACS, mitophagy, mKeima, Parkin, PINK1

## Abstract

Mitophagy is a selective autophagy process that specifically removes damaged mitochondria via general autophagy. The two major recessive Parkinson's disease genes PINK1 and Parkin play essential roles in mitophagy initiation, increasing the interest in mitophagy in both basic and translational research over the past 10 years. Initially, mitophagy was measured by the loss of mitochondria either through confocal imaging or immunoblot of mitochondrial proteins such as Tom20 or COXII. Confocal imaging of mitochondria DNA loss via anti‐DNA staining is another option. All of these methods, which take considerable effort and labor, are not sensitive enough to detect early stages of mitophagy with unambiguous objectivity. The mKeima assay can be used for both confocal imaging and FACS analysis to provide a thorough picture of mitophagy with a wide dynamic range. The Keima fluorophore has bimodal excitation under neutral and acidic pH conditions. Thus, when Keima is targeted to mitochondria it can accurately reveal the formation of mitolysosomes. Here the author briefly describes the origin and history of mKeima and how it is adapted to measure mitophagy. The author presents detailed protocols for making stable cell lines for optimized mitophagy detection and discuss many parameters that might affect the assay. A troubleshooting section is also provided to discuss possible pitfalls to improve reproducibility and sensitivity of the assay. © 2019 The Authors.

This article was corrected on 20 August 2022. See the end of the full text for details.

**Basic Protocol 1**: Making stable lines expressing mito‐mKeima and YFP‐Parkin

**Support Protocol**: Retrovirus/lentivirus infection

**Basic Protocol 2**: FACS analysis of mitophagy

## INTRODUCTION

**Figure 1:**
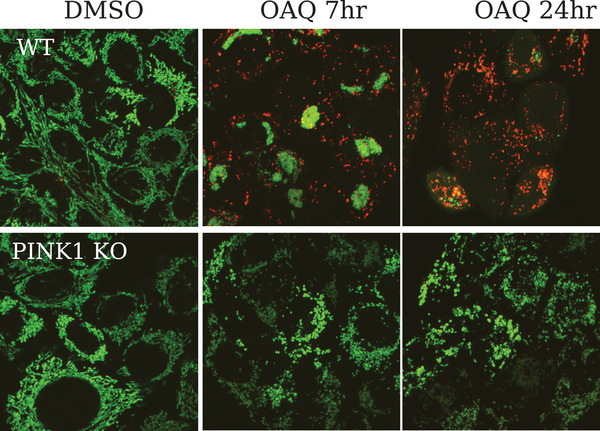
Confocal imaging of mitophagy with mito‐mKeima. WT or PINK1 KO HeLa cells stably expressing YFP‐Parkin (not shown) and mito‐mKeima were treated with dimethyl sulfoxide (DMSO) or oligomycin+Antimycin A+Q‐VD (OAQ) for 7 hr or 24 hr. mt‐mKeima excited at 488‐nm was artificially colored as green and mito‐mKeima excited at 561‐nm was artificially colored as red. In fact, mito‐mKeima emits at 620‐nm under either 488‐nm or 561‐nm excitation always appearing red under fluorescent microscope. Note that one drawback is that mito‐mKeima can only be observed in live cells as fixation will destroy the different pH environment required for differential mito‐mKeima excitation.

The purpose of this protocol is to provide readers with a detailed step‐by‐step instruction on how to measure mitophagy with a FACS‐based mKeima assay, which is extremely sensitive, reproducible, quantitative, objective and easy to perform. Although mitophagy has been extensively studied in many laboratories, it has been common practice, until recently, to use less ideal reporters such as degradation of mitochondrial outer membrane protein Tom20 for mitophagy studies without other supporting methods to corroborate their conclusions. Therefore, by establishing this simple mitophagy assay in more laboratories, a new standard method will be set to help clarify and verify some of the reports and facilitate reproducibility in mitophagy research. Complementing with confocal imaging (Fig. 1), the mKeima assay described here can provide a full picture of mitophagy dynamics at various time points.

In this method, the author first provide a basic protocol for how to establish stable cell lines expressing mito‐mKeima and YFP‐Parkin with either retrovirus or lentivirus. Although stable cell lines can be made with transient transfection followed by long‐term antibiotics selection or FACS sorting, virus infection is more efficient, stable and saves time in the long run. This protocol includes two parts: (1) How to package virus with a high titer. (2) How to infect cells with virus. In the second protocol, the author describes how to design and plan mitophagy experiments for FACS and how to perform FACS analysis to obtain the final data.

## Basic Protocol 1 GENERATION OF STABLE CELL LINES EXPRESSING MITO‐mKeima WITH VIRUS

Mito‐mKeima and YFP‐Parkin can be transiently transfected to monitor mitophagy. While this approach appears simple, it is less reproducible. Plasmid DNA quality, variation in transfection efficiency, and gene expression can all lead to uncontrolled alterations in performance. While producing stable cell lines requires more initial labor, they also provides undisputed benefits in terms of long‐term affordability and time requirements. Of note, although retrovirus or lentivirus transfer plasmids used here are defective in replication, they are still capable of integration. Therefore, caution should be taken when handling and disposing of the virus and associated materials.

Viral packaging is similar to regular transient transfection of an expression plasmid, with the only noteworthy difference being co‐transfection of helper plasmids. Afterwards, the virus can be easily harvested from the culture media and used to infect target cells. Targeted cells can be enriched by FACS sorting, as in this case, or selection (if proper anti‐biotic markers are present). For Parkin‐independent mitophagy, cells stably expressing only mito‐mKeima are required. However, it is recommended to make stable cells expressing both mito‐mKeima and YFP‐Parkin (or untagged Parkin) so that both Parkin‐mediated and Parkin‐independent mitophagy can be examined with different mitophagy inducers using the same stable cell line. A brief video of harvesting virus is provided below (Video 1).

### Materials

293T cells (ATCC #CRL‐3216)
Polyethylenimine, linear, MW 250000, transfection grade (PEI 25K; see recipe)
Opti‐MEM reduced serum medium (Thermofisher, cat. no. 31985062)
pCMV‐VSV‐G (Addgene, plasmid #8454)
pUMVC (Addgene, plasmid #8449)
pCHAC‐mt‐mKeima (Addgene, plasmid #72342)
pBMN‐YFP‐Parkin (Addgene, plasmid #59416)
pHDM‐G (Harvard Medical School, EvNO00061606)
pHDM‐HGPM2 (Harvard Medical School, EvNO00061607)
pHDM‐tat1B (Harvard Medical School, EvNO00061608)
pRC‐CMV‐rev1B (Harvard Medical School, EvNO00061616)
pHAGE‐mt‐mKeima (Addgene, plasmid #131626)
DMEM complete medium: DMEM medium (Thermofisher, cat. no.11960‐044 with phenol red or cat. no. 31053‐028) supplemented with 1× GlutaMAX (Thermofisher cat. no. 35050‐061),1 mM sodium pyruvate (Thermofisher, cat. no. 11360‐070), and 10% fetal bovine serum FBS), heat inactivated (Sigma, cat. no. F8317‐500 ml)

Nunc Cell Culture Treated 6‐well plates (Thermofisher, cat. no. 140675)
1.5‐ml MicroTube flat cap (SARSTEDT #72.690.300), autoclaved
Vortex mixer
Millex‐HV Syringe filter unit, 0.45‐µm, PVDF, 33‐mm, gamma sterilized (EMDmillipore, cat. no. SLHV033RB), ultra‐low protein binding
Monoject 1‐ml tuberculin syringe (Coviden, cat. no. 8881501400)
15‐ml polypropylene centrifuge tube (VitaScientific, cat. no. NSTF40173)

1 Seed 1–1.2 × 10^6^ 293T cells per well in a 6‐well plate.
  A variable number of 293T cells can be seeded. The key is to reach 70%‐90% confluency and allow the cells to attach well before transfection on the next day. It is ideal to seed the cells in the morning to allow cells to have enough time to attach. 293T cells do not adhere well and use caution when handling the cells to prevent detachment. Exercise close attention on maintaining 293T cells to ensure they remain flat and spread out. Cells normally look “clumpy” 1 day after thawing. If the clumping of the cells persists, passage cells a few more times to achieve the correct appearance. Cells should not be split too thin (less than 1:10 split), as this will promote clumping and will be slow to recover back to a normal morphology.
2 Perform transfection the next day in the afternoon with PEI 25K as in steps 3‐8.
  Any commercial transfection reagents can be used for transfection. PEI 25K is the cheapest and provides high transfection efficiency.
3 Add 0.5 ml Opti‐MEM into a sterile 1.5‐ml microcentrifuge tube.
4 For retrovirus, add 0.5 µg pCMV‐VSV‐G, 1 µg pUMVC, 1.5 µg pCHAC‐mt‐mKeima or pBMN‐YFP‐Parkin.
5 For lentivirus, add 0.5 µg of each helper plasmid: pHDM‐G, pHDM‐HGPM2, pHDM‐tat1B, pRC‐CMV‐rev1B and 1 µg pHAGE‐mt‐mKeima.
  One can use other retrovirus or lentivirus helper plasmids and the ratio between the helper plasmids and the virus transfer plasmid may be altered to achieve the highest titer. The helper plasmids can also be pre‐mixed to reduce pipetting steps.
6 Add 9 µl of PEI 25K. Vortex for 15‐30 s. Let sit for 15 to 30 min at room temperature.
7 Replace the medium from the 6‐well plate with 1.5 ml fresh DMEM complete medium.
  Although the original PEI transfection protocol requires DMEM medium containing only 1% FBS, we observed that normal DMEM medium containing 10% FBS can be used without affecting transfection efficiency and virus titer.
8 Add the transfection mix drop wise to the cells. Gently swirl the plate to mix as 293T cells detach easily. Gently swirl while also ensuring a proper mixing.
9 Change the medium the following day with 1 ml fresh DMEM complete medium.
  When changing the medium, caution should also be taken. Add the medium along the side of the well to avoid disturbing the cells.
10 Harvest the virus 2 days after transfection. Remove the medium containing the virus from the 6‐well plate and put into a 1.5‐ml sterile microcentrifuge tube.
11 Quickly spin for 1 min at 1000 × *g* to pellet any carry‐over cells or debris.
12 Transfer the supernatant into a new sterile 1.5‐ml microcentrifuge tube. Virus can be used right away or stored up to 1 month at 4°C and for long‐term at −80°C.
  Simply centrifuging the tubes to remove any carry‐over of 293T cells is not always complete. The following filtration step is highly recommended to ensure no contamination of 293T cells.
13 To filter the virus, use 0.5‐1 ml DMEM complete medium to pre‐wet the PVDF Millex‐HV filter with a 1‐ml syringe, and then filter the medium containing virus (step 10) into a sterile 15‐ml centrifuge tube (see Video 1).
  NOTE: All pipette tips, tubes, filters and plates that came in touch with virus should be secured in a sealed bag and then placed in the appropriate biological waste can. Solutions or media containing virus should be put into a bottle with 10% bleach.

## RETROVIRUS/LENTIVIRUS INFECTION

Virus transduction/infection is a straightforward method to generate stable cell lines. We recommend omitting the measurement of virus titer since MOI (multiplicity of infection) is not always a good indicator of transduction rate and proper gene expression. Instead, the expression level of the proteins of interest after transduction should be used as the benchmark for virus quality. It normally takes 2 days for genes transduced to be expressed and 3 days for maximal expression. If the fluorescent signal is weak or moderate 2 days after infection, this is the sign that the virus titer used is sufficient. If the signal is still too weak on Day 3, then re‐infection can be performed. It is preferred not to infect cells with too much virus since very bright mKeima or YFP signal can be problematic for FACS analysis as the two fluorescent signals could interfere with each other.

The binding of virus particles to target cells is the key determining factor for a higher infection rate. This initial binding step was assumed to involve specific interactions between the viral envelope and specific receptors on the cell surface but was later shown to be receptor‐independent. Subsequent strong electrostatic repulsion between the negatively charged cells and approaching enveloped virus counteracts the binding of the viral envelope to the cells. Low speed centrifugation or addition of positively‐charged polycations such as polybrene, reduces the repulsive forces and greatly enhances the transduction efficiency. The optimal concentration of polybrene can be cell‐type specific and certain cells such as neurons are very sensitive to polybrene.

### Additional Materials (also see Basic Protocol 1)

Target cells
Polybrene (see recipe)
Retrovirus *or* lentivirus (see Basic Protocol 1)
Fluorescent microscope

1 Seed target cells in a 6‐well plate so that cells are at 10%‐20% confluency the following day. For HeLa cells, seed 5K‐10K cells per well.
  Depending on the virus titer, higher infection rate can be achieved by seeding less cells. Generally, lentivirus produces a higher titer than retrovirus. However, retrovirus tends to produce more uniform gene expression.
2 In a 15‐ml centrifuge tube, add 4 µl Polybrene (500×), 0.5 ml retrovirus or 0.2‐0.3 ml lentivirus, culture medium up to 2 ml. Gently mix by pipetting.
  Virus volume should not exceed half of the final infection volume as inhibitory factors can be produced during virus packaging. If frozen virus is used, thaw virus quickly in a 37°C water bath.
3 Remove the culture medium from the target cells.
4 Add virus mix to the cells.
5 Virus can be removed the next day from the cells
  Sometimes virus with a higher titer can be toxic and should not be left in the cells for extended periods of time. Check cell behavior the following day after infection. If there are no signs of toxicity, media containing virus can be left for longer.
6 Check mito‐mKeima or YFP‐Parkin signal under a fluorescent microscope on Day 2 after infection
  If the fluorescent signal is faint or moderate on Day 2, it is a good indication that mito‐mKeima or YFP‐Parkin are expressed at the proper level. Less virus should be used if mKeima or YFP signal is too high.
7 Mito‐mKeima or YFP‐Parkin signal should be maxima 3 days after infection and cells are ready for further experiments.
  If a cell population with even expression of mKeima and YFP is required, cells can be sorted by FACS to enrich for the double‐positive cells with the desired expression level.

## Basic Protocol 2 FACS ANALYSIS OF MITOPHAGY

In their original paper reporting the use of mKeima for autophagy and mitophagy measurement, Katayama et al. described an ratiometric imaging‐based approach, which is very limited on the number of cells (30 transfected cells calculated) that could be imaged and analyzed. As shown in Figure 1, mito‐mKeima puncta, due to its localization in the matrix of mitochondria, appear larger and distinct during mitophagy comparing to mKeima puncta during autophagy. Some researches simply count the number of red‐only mito‐mKeima puncta or mito‐QC and other similar fluorescent reporters for mitophagy measurement. This is clearly not accurate and likely overestimate the extent of mitophagy. Ratiometric imaging of mito‐mKeima seems to reflect the overall mitophagy better but is time consuming.

We decided to explore the usage of FACS for easier, quantitative and more objective measurement of mitophagy, which turn out to be very successful. Now, 50,000 cells instead of 30 cells can be analyzed. A step‐by‐step video demo of how to use FlowJo software for FACS analysis covering steps 10‐12 is provided below (see Video 2).

### Additional Materials (also see Basic Protocol 1)

Antimycin A (Sigma, cat. no. A8674): Dissolve in 100% ethanol to make 8 mg/ml stock (2000×)
Oligomycin (mixture of A, B, C isomers) (Millipore, cat. no. 495455): Dissolve in dimethyl sulfoxide (DMSO) to make 10 mg/ml stock (1000×)
Q‐VD (ApexBio, cat. no. A1901): Dissolve in DMSO to make 10 mM stock (1000×)
Hanks’ balanced salt solution (HBSS; Thermofisher, cat. no.14175‐095)
0.05% Trypsin‐EDTA (1×) (Thermofisher, cat. no.25300‐054)
DAPI (diamidino‐2‐phenylindole), 1 mg/ml solution (10,000×) (Thermofisher, cat. no.62248)

Costar 12‐well clear TC‐treated multiple well plates (Corning, cat. no. 3512)
Benchtop centrifuge (Eppendorf 5424)
Falcon 5‐ml polystyrene round‐bottom tube with cell‐strainer cap (Corning, cat. no. 352235)
MoFlo Astrios cell sorter (Beckman Coulter)
Summit software (v6.2.6.16198)
FlowJo software

**Figure 2:**
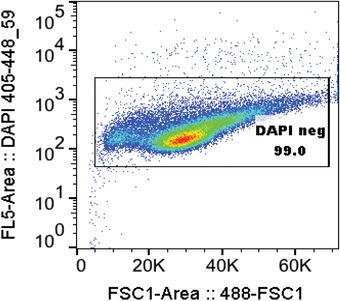
Gating live cells with the aid of DAPI.

1 Seed 100K HeLa cells stably expressing YFP‐Parkin and mito‐mKeima per well in 12‐well plates.
  If a 6‐hr time point is needed, seed 150K cells. Seed appropriate number of cells depending on the duration of experimental treatments so that cells do not become overconfluent during the duration of the experiment.
2 Treat the cells with OAQ (diluted from 1000×−2000× stocks of each chemical) for 6‐24 hr at 37°C.
  Oligomycin coupled with antimycin A induces mitochondria membrane potential loss and triggers robust Parkin‐mediated mitophagy. Q‐VD, a potent apoptosis inhibitor, is used to prevent cell death during this process.
3 Wash the cells with 0.5 ml HBSS buffer
4 Trypsinize the cells with 0.2‐0.3 ml 0.05% trypsin‐EDTA. Incubate at 37°C for a couple of minutes until cells dislodge from the plate.
5 Add 0.5 ml DMEM culture medium to the wells. Mix and transfer the cells into a 1.5‐ml microcentrifuge tube.
  At this step, non‐sterile centrifuge tubes can be used.
6 Centrifuge for 1 min at 300‐1000 × *g* to pellet the cells.
  Higher speed and duration of centrifugation is not recommended as this will cause mechanical sheering of the cells and also make it difficult to resuspend the cells.
7 Remove the medium and resuspend the pellet in 0.3 ml DMEM culture medium containing 100 ng/ml DAPI (diluted from the 10,000× stock).
  DAPI is used for staining dead cells allowing for gating of live cells only during FACS. It is not necessary but is recommended. Cells should be resuspended well to avoid any clumps that may clog the machine.
8 Transfer the cells into 5 ml round‐bottom tube with cell‐strainer cap.
  Cell strainer will help to remove cell clumps that could clog the machines during FACS analysis.
9 Bring the samples to the FACS facility for analysis.
10 With MoFlo Astrios cell sorter, for each sample, 50k‐100k events should be collected and first gated for live cells (DAPI‐negative) (See Fig. 2, Video 2):
  - Y‐axis: DAPI height or area log 355_488‐59 or log 405_448‐59
  - X‐axis: FSC height or area log 488
  - Use gate from control sample containing all 4 populations for all proceeding samples.

**Figure 3:**
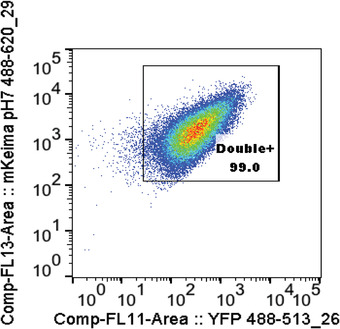
Gating single (mt‐mKeima) or double positive (mt‐mKeima and YFP‐Parkin) cells.

11 Compensate on DAPI‐negative cells (See Fig. 3, Video 2):
  - Y‐axis: mKeima pH 7 488_620‐29 area log compensation
  - X‐axis: YFP 488_513‐26 area log compensation
  - Gate double‐positive cells for control sample containing all 4 populations and then use this same gate for all proceeding samples.

**Figure 4:**
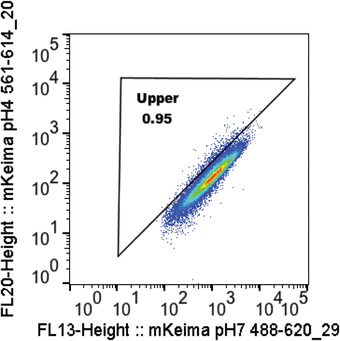
Ratiometric analysis.

12 Ratiometric analysis (See Fig. 4, Video 2):
  - Y‐axis: mKeima pH 4 561_614‐20 area log (or height)
  - X‐axis: mKeima pH 7 488_620‐29 area log (or height)
  - Draw upper and lower gates using WT and Pink1 KO as positive and negative controls, respectively, then apply the same gates for all proceeding samples.

## REAGENTS AND SOLUTIONS

### PEI 25K stock, 1 mg/ml

Prepare PEI (Polysciences, cat. no. 23966‐1) in 20 mM HEPES, which may take up to 2 hr at room temperature to dissolve completely. Heating up to 80°C can facilitate dissolving. Filter the stock through a 0.22‐µm filter, divided into 0.2‐0.5‐ml aliquots and store up to 6 months at −80°C. After thawing, the rest of PEI can be stored at 4°C for a couple of weeks. If notable drop of transfection efficiency occurs, remake the PEI stock fresh every 4‐6 months.

### Polybrene stock, 4 mg/ml

Prepare polybrene (Hexadimethrine bromide, Sigma, cat. no. H9268) in 1× phosphate‐buffered saline (PBS) and filter through a 0.22‐µm filter. Store long‐term at −20°C. For convenience, it can be stored for a couple of months at 4°C.

## COMMENTARY

### Background Information

Mitophagy is a type of selective autophagy event that specifically removes damaged and dysfunctional mitochondria caused by accumulation of mitochondrial DNA (mtDNA) mutations or misfolded proteins, loss of mitochondrial membrane potential, and production of reactive oxygen species (ROS). Seminal work by Narendra et al. (2010) discovered that PINK1 kinase, which is normally degraded via the N‐end rule under normal conditions (Yamano & Youle, 2013), is stabilized and activated on damaged mitochondria. PINK1 activation triggers phosphorylation of both ubiquitin and Parkin, a E3‐ligase, which promotes Parkin to translocate to damaged mitochondria and ubiquitinate many mitochondrial outer membrane proteins. Ubiquitin chains on mitochondrial proteins are bound by autophagy adaptors such as NDP52, OPTN, and TAX1BP1 to mitochondria, which in turn bind and recruit the general autophagy machinery to mitochondria to initiate the mitophagy.

Initially, the mitochondrial outer membrane protein Tom20 was used to monitor the clearance of mitochondria by confocal imaging or Western blot (Narendra, Tanaka, Suen, & Youle, 2008). Later on, it was revealed that many mitochondrial outer membrane proteins that are ubiquitinated by Parkin undergo ubiquitin/proteasome system (UPS)‐mediated degradation rather than autophagic degradation (Chan et al., 2011; Sarraf et al., 2013; Tanaka et al., 2010), making them unsuitable for monitoring mitophagy.

One of the widely used mitophagy reporters, mito‐QC, which mimics the GFP‐RFP‐LC3 for autophagy flux, was created by fusing mCherry‐GFP in tandem with the C‐terminal tail (101‐152) of the outer membrane protein Fis1 (Allen, Toth, James, & Ganley, 2013). Under normal conditions, mito‐QC fluoresces both red and green to appear yellow together. Upon induction of mitophagy, mitochondria are engulfed by autophagsosomes, which then fuse with lysosomes to form autolysosomes. In this acidic environment, the GFP signal gets quenched but mCherry signal remains stable to make mito‐QC appear to be red‐only. The percentage of red‐only mito‐QC signal can be used to measure mitophagy. As mentioned above, mito‐QC itself can be degraded at least partially via the UPS and may result in an underestimation of the mitophagy events. In addition, factors involved in the UPS pathway may inadvertently influence mito‐QC readings.

mKeima belongs to a class of fluorescent proteins with large Stokes shifts (the difference in wavelength between the peak excitation and emission spectra). It was originally identified from the stony coral *Montipora* sp. with degenerate primers and semi‐random mutagenesis (Kogure et al., 2006). The first identified clone was a violet‐colored GFP‐like chromoprotein that did not fluoresce. However, a red fluorescent protein was successfully created with five substitutions (H94N, N142S, N157D, K202R and F206S) and the addition of a valine residue at the second amino‐acid position. Subsequently, four more mutations (S61F, I92T, F158Y, and S213A) were introduced to generate the Keima protein, a shogi (Japanese chess) piece that hops in the manner of the knight in chess, owing to the large Stokes shift. Keima shows a bimodal excitation spectrum peaking at 440 and 580 nm and emission spectrum peaking at 606 nm. This Keima is homotetrameric, which was further converted into a dimeric dKeima with introduction of V123T and V191I mutations. Additional mutations (L60Q, F61L, V79F, T92S, T123E, Y188R, and Y190E) led to the monomeric mKeima protein, which now shows maximal emission at 620 nm. The two excitation peaks somehow correspond to the neutral and acidic pH. Violot, Carpentier, Blanchoin, and Bourgeois (2009) used X‐ray crystallography to discover that the large Stokes shift of mKeima is due to the reverse pH‐dependence of chromophore protonation.

Katayama, Kogure, Mizushima, Yoshimori, and Miiyawaki (2011) first reported the use of mKeima and mito‐mKeima for autophagy and mitophagy measurements. They showed that mKeima has a p*K*_a_ value of 6.5 and excitation peak ratio (586/440) of more than 6. Unlike mito‐QC, mito‐mKeima can be used together with GFP/YFP tagged proteins. By measuring the proportion of the high ratio (550/438) signal area to the total mitochondria area as an index of mitophagy (IM) in 30 transfected cells, IM of 60% can be detected in MEF cells treated with CCCP + oligomycin. There is an obvious limitation with this imaging‐based method as only 30 cells are examined. To improve the speed and sensitivity, we explored the possibility of using flow cytometry (FACS) to quantitatively and quickly measure mitophagy with mito‐mKeima (Lazarou et al., 2015). We successfully detected as low as 5% mitophagy events when PINK1 was artificially tethered on mitochondria via chemically‐induced dimerization, which is crucial to the newly proposed model: PINK1‐generated phosphor‐ubiquitin itself serves as the mitophagy signal and Parkin is a mere amplifier of this signal. By using FACS to detect mitophagy, over 50,000‐100,000 events can be collected and analyzed, which increases the detection threshold of mitophagic events for better data representation.

### Critical Parameters

For viral packaging, the most critical factor is the health and behavior of 293T cells, as transfection efficiency is rarely an issue for them. 293T cells must be split regularly, preferably at 1:5 ratio and with increased attention on how they attach and spread. Right after thawing, especially when seeded at a low density, 293T cells tend to form clumps.

We tried several different transfection reagents and did not observe any difference in virus titer when similar transfection efficiency was obtained. Occasionally, we encountered an issue of low virus titer and traced the problem back to the helper plasmids. Some of the helper plasmids have the potential to rearrange, causing small deletions, which can be detected by agarose gel electrophoresis even without digestion. We solved the problem by re‐transforming the correct plasmids in NEB or Thermofisher's Stable competent cells. Therefore, it is very important to retain original helper plasmid stocks either in bacteria or in solution as backup for future amplification.

In terms of transduction or infection, forgetting to add polybrene is the most likely cause of low transduction rate, followed closely by over‐seeding too many cells for infection. Freeze‐thaw cycles of viruses can lead to 5‐fold reduction in virus titer. We found virus can be stored for at least 1 month at 4°C without losing significant titer. Otherwise, virus should be divided into aliquots and kept for long‐term storage at −80°C. When thawing the virus, the frozen virus should be incubated for a few minutes at 37°C and used quickly. Furthermore, the addition of too much virus with a high titer could be toxic. Sometimes, culture medium for target cells differ from medium for 293T cells. In this case, virus volume should be at least less than half of the final medium volume for infection.

For mitophagy assays, the most crucial element is to have proper controls. With FACS analysis, DAPI, or DRAQ7 (far‐red) should be used to gate out the dead cells. When gating either the mKeima or YFP channel, compensation might be needed to avoid interference between each channel. This is often the issue when either mKeima or YFP signal is too high. Reducing laser power could help to limit the interference but sometimes still does not solve the problem. Therefore, it is always better to do a pilot experiment to find out the optimal virus titer for infection. It is better to begin with a weak signal, as re‐infection can be done to enhance the signal. For the final gating of mKeima ratio (pH4, 561 nm/pH7 488 nm), always re‐normalize the untreated control of each sample set to around 1%. This is because different cell lines may have different basal mitophagy level to begin with. In addition, mKeima and YFP intensity are not identical in different cell lines even after sorting to obtain similar intensity level. Therefore, applying the same mKeima ratio gating to different cell lines is not practical and also not advised.

### Troubleshooting

For possible problems and their solutions, see Table 1.

**Table 1:** Troubleshooting Problems and Possible Solutions

Problem	Solution
Low virus titer	Make sure 293T cells never grow confluent, split when they reach 80%‐90% confluence. Sometimes, splitting cells several times after thawing may help, too.
	Seed cells so that they are 70%‐90% confluent the day before transfection. Make sure cells adhere well and spread nicely. Do not attempt transfection if cells are clumpy.
	293T cells do not attach well, so be gentle when moving plates in and out the incubator. After transfection mix is added to the cells, swirl the plates very gently a couple of times to mix so that cells do not get dispersed and come off the plates.
	Check the size of helper plasmids. Some helper plasmids tend to undergo recombination and small deletions might occur, which can be detected on agarose gels. Transform the helper plasmids in NEB's Stable cells (NEB, cat. no. C3040I) or similar competent cells defective in recombination for mini or midi prep.
Low transduction rate	Seed cells so that they are less than 20% confluent on the day of infection. 5%‐10% confluent could be used if virus titer is not high.
	Make sure polybrene is added during infection. 4‐8 µg/ml works well for most cell lines. Some cell lines are sensitive and lower polybrene concentration has to be used if cells die quickly after infection.
	Use more virus.
mito‐mKeima or YFP‐Parkin signal is not even	Retrovirus seems to produce more even gene expression than lentivirus. If an even signal is needed, FACS sort the cells
Mitophagy in wild type cells is not robust	Different cell lines may require different concentrations of oligomycin, antimycin A for robust mitophagy response. Treatment time can also be a factor. Make sure fresh chemicals are used since they may lose activity over time or not properly stored.
	Parkin expression is too low. While robust mitophagy does not require maximal Parkin expression, detectable Parkin expression level is indeed needed. It is ideal to use YFP‐Parkin so that Parkin expression level can be readily monitored. Untagged or HA‐Parkin can be used when YFP‐Parkin is not an option.

### Understanding Results

**Figure 5:**
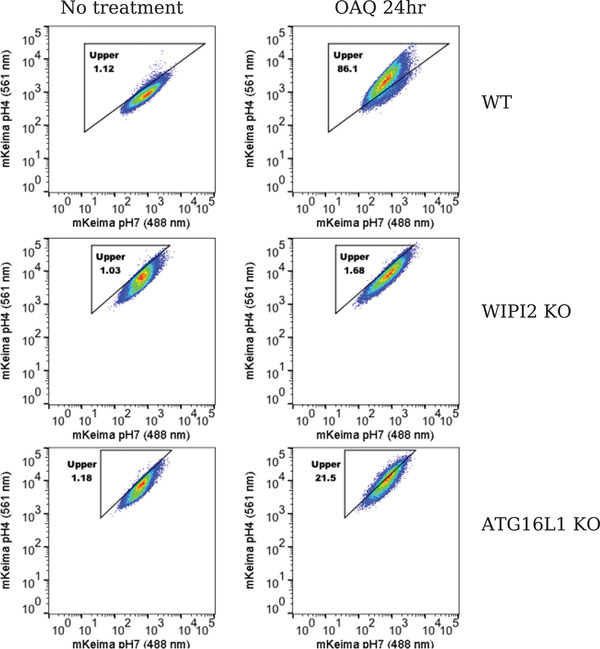
An example of mitophagy measurement with FACS analysis. The same exact gating was used for each pair of samples (treated vs. untreated).

Anticipated results are shown in Figure 5. In untreated wild‐type HeLa cells, mitophagy (shown as the upper box) is gated to around 1%. With OAQ treatment for 24 hr, 86.1% mitophagy is detected. In WIPI2 KO cells, mitophagy is gated to around 1% in the untreated cells, only 1.68% mitophagy is observed after 24 hr of OAQ treatment, indicating that WIPI2 KO completely blocks Parkin‐mediated mitophagy. In ATG16L1 KO cells, 21.5% mitophagy is seen with OAQ treatment for 24 hr, comparing to 1.18% in untreated cells, suggesting that ATG16L1 strongly inhibits Parkin‐mediated mitophagy. Different cell lines can have different cell size and mKeima signal, which reflects the different distribution patterns of cell population. When the triangle box is drawn to delineate the upper portion (mitophagy), make sure the diagonal line is parallel to the red/orange area (peak of the cell population) in the untreated control cells. See the video for details (Video 2).

### Time Considerations

It takes 3‐4 days to package virus from plating 293T cells for transfection to harvest virus on Day 2 and Day 3. For transduction/infection, 2‐3 days are also required from seeding target cells to see optimal gene expression. For FACS analysis or confocal imaging, depending on the treatment time, 2‐3 days should be planned.

## Corrections

In this publication, the following corrections have been made.

Page 4, step 5, first line: “1 µg of each helper plasmid” has been replaced with the correct amount.

The current version online now includes these corrections and may be considered the authoritative version of record.

## Supporting information

??Supporting Information 1??

??Supporting Information 2??
